# LC-MS/MS Confirms That COX-1 Drives Vascular Prostacyclin Whilst Gene Expression Pattern Reveals Non-Vascular Sites of COX-2 Expression

**DOI:** 10.1371/journal.pone.0069524

**Published:** 2013-07-09

**Authors:** Nicholas S. Kirkby, Anne K. Zaiss, Paula Urquhart, Jing Jiao, Philip J. Austin, Malak Al-Yamani, Martina H. Lundberg, Louise S. MacKenzie, Timothy D. Warner, Anna Nicolaou, Harvey R. Herschman, Jane A. Mitchell

**Affiliations:** 1 National Heart & Lung Institute, Imperial College London, London, United Kingdom; 2 The William Harvey Research Institute, Barts & the London School of Medicine & Dentistry, Queen Mary University of London, London, United Kingdom; 3 Department of Molecular and Medical Pharmacology, University of California Los Angeles, Los Angeles, California, United States of America; 4 School of Pharmacy, University of Bradford, Bradford, United Kingdom; 5 King Fahad Cardiac Center of King Saud University, Riyadh, Saudi Arabia; 6 School of Life and Medical Sciences, University of Hertfordshire, Hertfordshire, United Kingdom; McMaster University, Canada

## Abstract

There are two schools of thought regarding the cyclooxygenase (COX) isoform active in the vasculature. Using urinary prostacyclin markers some groups have proposed that vascular COX-2 drives prostacyclin release. In contrast, we and others have found that COX-1, not COX-2, is responsible for vascular prostacyclin production. Our experiments have relied on immunoassays to detect the prostacyclin breakdown product, 6-keto-PGF_1α_ and antibodies to detect COX-2 protein. Whilst these are standard approaches, used by many laboratories, antibody-based techniques are inherently indirect and have been criticized as limiting the conclusions that can be drawn. To address this question, we measured production of prostanoids, including 6-keto-PGF_1α_, by isolated vessels and in the circulation *in vivo* using liquid chromatography tandem mass spectrometry and found values essentially identical to those obtained by immunoassay. In addition, we determined expression from the *Cox2* gene using a knockin reporter mouse in which luciferase activity reflects *Cox2* gene expression. Using this we confirm the aorta to be essentially devoid of *Cox2* driven expression. In contrast, thymus, renal medulla, and regions of the brain and gut expressed substantial levels of luciferase activity, which correlated well with COX-2-dependent prostanoid production. These data are consistent with the conclusion that COX-1 drives vascular prostacyclin release and puts the sparse expression of *Cox2* in the vasculature in the context of the rest of the body. In doing so, we have identified the thymus, gut, brain and other tissues as target organs for consideration in developing a new understanding of how COX-2 protects the cardiovascular system.

## Introduction

Prostacyclin, a powerful cardioprotective hormone released by the vascular endothelium, inhibits platelet activation, vascular remodeling and atherosclerosis. Consequently, inhibition of prostacyclin release has been associated with an increased risk of heart attacks and strokes [1]. Prostacyclin production results from the consecutive actions first of cyclooxygenase (COX), which converts arachidonic acid to prostaglandin (PG) H_2_, the precursor of all prostanoids, followed by the action of prostacyclin synthase, which isomerizes PGH_2_ to mature prostacyclin.

Two COX isoforms exist; COX-1 and COX-2 [2–4]. COX-1 is expressed constitutively in many tissues [5,6]. COX-2 expression, in contrast, is normally sparse in most tissues but is rapidly upregulated by mitogens, cytokines and other stimuli; COX-2 dependent prostanoids contribute to cell proliferation, pain and inflammatory responses [7,8]. Traditional non-steroidal anti-inflammatory drugs (NSAIDs), such as ibuprofen and diclofenac inhibit both COX-1 and COX-2 isoforms. Much of the analgesic and anti-inflammatory benefit of these agents is derived from inhibition of COX-2, whilst concurrent inhibition of COX-1 produces unwanted and potentially life threatening gastrointestinal side effects [9]. Consequently, new COX-2 selective agents such as celecoxib (Celebrex^TM^) and rofecoxib (Vioxx^TM^) have a reduced incidence of gastrointestinal side effects, while retaining anti-inflammatory and analgesic efficacy [10]. It is now clear that both traditional NSAIDs and COX-2 selective inhibitors are also associated with a small but definite increase in the risk of atherothrombotic events in man [11], particularly myocardial infarction. These clinical data are consistent with data from animal models demonstrating that either global *Cox2* gene deletion or global pharmacologic COX-2 enzyme inhibition produce a pro-atherogenic, pro-thrombotic phenotype [12–15].

With regard to the cardiovascular system and particularly the vascular endothelium, there has been strong debate regarding which COX isoform is predominant and responsible for prostacyclin production. Opinion is divided, with two opposing views. It is currently widely held that COX-2 expression and activity predominates over COX-1 within endothelial cells and consequently is the major driver of vascular prostacyclin production [1,14–16]. Inhibition of COX-2-dependent production of cardioprotective prostacyclin in the cardiovascular endothelium has been proposed to explain the increase in cardiovascular events observed in patients taking both traditional and COX-2-selective NSAIDs [13,16]. This hypothesis is rooted in studies showing that urinary excretion of prostacyclin markers are reduced in human volunteers receiving COX-2 inhibitors [17], mice that have a global *Cox2* gene deletion [5,12], and mice that have targeted endothelial and/or vascular smooth muscle *Cox2* gene deletions [14]. The suggestion that inhibition of COX-2-dependent vascular prostacyclin synthesis is responsible for the increased cardiovascular events is further supported by the atherothrombotic phenotype of *Cox2* [12–14] and prostacyclin receptor [18] knockout mice, consistent with this hypothesis.

Whilst not all investigators find urinary prostacyclin markers to be reduced in global *Cox2* gene knock out mice [19], recent data from our group support this idea [5]. However, we found that urinary markers do not to reflect prostanoid formation in the vasculature [5], suggesting instead that they may reflect more localized prostacyclin production, perhaps in the kidney by blood vessels of the vasa recta, where COX-2 is constitutively expressed [20]. Thus, in direct contrast to the commonly accepted hypothesis, work from our group [5] and others [21] demonstrates that COX-1 is the dominant isoform in the vascular endothelium driving prostacyclin production.

Our work and that of others in this area has routinely relied on the use of immunoassays to detect COX products [22–26] and the use of antibodies to detect COX-1 and COX-2 protein expression in tissues [5,14]. Whilst these techniques to measure prostanoids and proteins are standard practice, the use of antibodies for detection of any product is inherently indirect and, as was recently highlighted [16], open to artifact. Primarily based on these two objections, our conclusion that COX-1 drives vascular prostacyclin has been challenged [16].

In addition to the above concerns, we note that our previous studies focus on the role of COX-2 in vascular prostacyclin production; they were not designed to consider other sites of COX-2 expression, or the effect of loss of COX-2 activity on prostanoids other than prostacyclin. In the current study we perform new experiments to directly address these methodological and biological limitations. Firstly, we validate our conclusions regarding prostanoid production, drawn previously from immunoassay studies, by employing liquid chromatography tandem mass spectrometry (LC-MS/MS) to assess lipid mediator release both from isolated vessels and in the circulation *in vivo*, profiling the effect of global *Cox2* gene deletion on a range of prostanoid metabolites. Secondly, we employed a reporter mouse in which the luciferase coding region is knocked into the *Cox2* gene, and is thus under *Cox2* gene regulatory control [27], to directly visualize, quantitate and compare expression from the *Cox2* gene in the regions of the vasculature as well as a panel of other tissues. Use of *Cox2* promoter driven luciferase expression eliminates the requirement for antibody evaluation of expression from the *Cox2* gene. Together these studies support our previous observations that COX-1, not COX-2, drives prostacyclin release in the vasculature, and provide much needed new targets for understanding how COX-2 inhibition might regulate cardiovascular function.

## Materials and Methods

### Mice

Cox1^-/-^ [28], Cox2^-/-^ [28] and *Cox2* ^*fLuc/+*^ mice [27] were generated as previously described, and back-crossed onto a C57Bl/6J background. Wild-type mice were generated by inter-crossing C57Bl/6 back-crossed Cox1^+/-^ and Cox2^+/-^ mice. All mice used in the study were genotyped before use. Experiments were performed on male and female mice at 10-12 weeks old. Animal procedures were conducted in strict accordance with Animals (Scientific Procedures) Act 1986 and the recommendations in the Guide for the Care and Use of Laboratory Animals of the National Institutes of Health. Protocols were subject to local ethical review and approval by the Imperial College Ethical Review Panel (PPL No. 70/7013) or the UCLA Animal Research Committee (Protocol. No. 1999-066-43; luciferase imaging experiments only). All surgical procedures and luciferin treatments were performed under isoflurane anesthesia, taking all appropriate measures to minimize suffering. *Ex vivo* and *in vitro* experiments were performed on tissue removed from humanely euthanized animals (see details below).

### In vitro COX activity bioassays

Wild-type, *Cox1* ^*-/-*^ and *Cox2* ^*-/-*^ were euthanized by CO_2_ narcosis and the vasculature perfused with PBS. Aortic tissue and various solid tissues were carefully dissected into small pieces (~2mm rings for aortic tissue, ~25mm^3^ for solid organs) and immediately placed into individual wells of 48 or 96 well microtitre plates containing Ca^2+^ ionophore A23187 (50µM; Sigma, UK) in DMEM (+200mM L-Glutamine; Sigma, UK). Tissues were incubated for 30 mins at 37°C, before collection of the supernatant to measure prostanoid release by immunoassay or LC-MS/MS. For studies of relative release by different tissues, prostanoid release was normalized to tissue wet weight.

### Circulating prostanoid measurement in vivo

Under isoflurane anesthesia, the right jugular vein and left carotid artery of wild-type, Cox1^-/-^ and Cox2^-/-^ mice were cannulated. After a 20 min stabilization period, bradykinin (100nmol/kg; Tocris Bioscience, UK) was administered intravenously and 0.8ml arterial blood collected 5 mins later in to heparin (10U/ml final; Leo Laboratories, UK). After blood collection, animals were immediately euthanized by cervical dislocation without being allowed to recover from anaesthetic. Plasma was separated from blood by centrifugation and the levels of prostanoids measured by LC-MS/MS.

### Bioluminescent imaging

*Cox2* ^*fLuc/+*^ mice were injected intraperitoneally with D-luciferin (125 mg/kg, Xenogen, USA) under light isoflurane anesthesia and 15 mins later euthanized by overdose the same anesthetic. Tissues were rapidly dissected and placed in culture dishes. Bioluminescent emission was recorded over 3 mins, using the IVIS imaging system (Xenogen, USA). Collected photon number and images were analyzed using Living Image software (Xenogen, USA) and quantified as the peak photon release/pixel detected from each tissue.

### Luciferase activity

After bioluminescent imaging, tissues were snap frozen for biochemical measurement of luciferase activity using the Luciferase Assay System (Promega, UK). Tissues were dissociated using a Precellys24 bead homogenizer in passive lysis buffer (Promega, UK) and loaded into white 96 well microtitre plates. The time-integrated (10 sec) luminescence of each well was then read, 15 secs after injection of 10X volume of Luciferase Assay Reagent (Promega, UK). Protein concentration of homogenates was determined using the bicinchoninic acid method (Perbio, UK) and used to normalize luciferase activity data.

### Prostanoid immunoassays

In some experiments, the stable prostacyclin breakdown product 6-keto-PGF_1α_ was measured using either a competitive immunoassay kit (Cayman Chemical, USA), or where noted and in separate biological samples, by radioimmunoassay using 6-keto-PGF_1α_ antisera (Sigma, UK) and [^3^H] 6-keto-PGF_1α_ (Amersham Biosciences, UK). PGE_2_ was measured using a commercially available homogenous time-resolved fluorescence-based immunoassay (Cisbio, France).

### Prostanoid measurement by LC-MS/MS

Prostanoids were extracted and analyzed as previously described [29]. Briefly, 400-500 l sample was mixed with 3ml ice-cold 15% methanol (v/v) and PGB_2_-*d4* (40 ng) was added as internal standard. The samples were then acidified to pH 3.0 and the prostanoids were semi-purified using solid phase extraction (Phenomenex, UK). LC-MS/MS of the lipid extract was performed on a triple quadrupole mass spectrometer equipped with an electrospray probe and coupled to liquid chromatography (Waters, UK). Analysis of prostanoids was based on MRM assays using the following transitions: 6-keto PGF_1α_ *m*/*z* 369>163; PGE_2_ *m*/*z* 351>271; 13, 14-dihydro 15-keto PGE_2_ *m*/*z* 351>333; PGD_2_: *m*/*z* 351>271; TXB_2_ *m*/*z* 369>169; PGF_2__α_ *m*/*z* 353>193; PGB_2_-*d4*: *m*/*z* 337>179. Results are expressed as pg metabolite / ml plasma or culture medium, using calibration lines constructed with commercially available prostanoid standards (Cayman Chemicals, USA).

## Results and Discussion

### Role of COX-1 and/or COX-2 in prostacyclin release by vessels in vitro; measurement of 6-keto-PGF_1α_ with immunoassays and mass spectrometry

**Figure 1:**
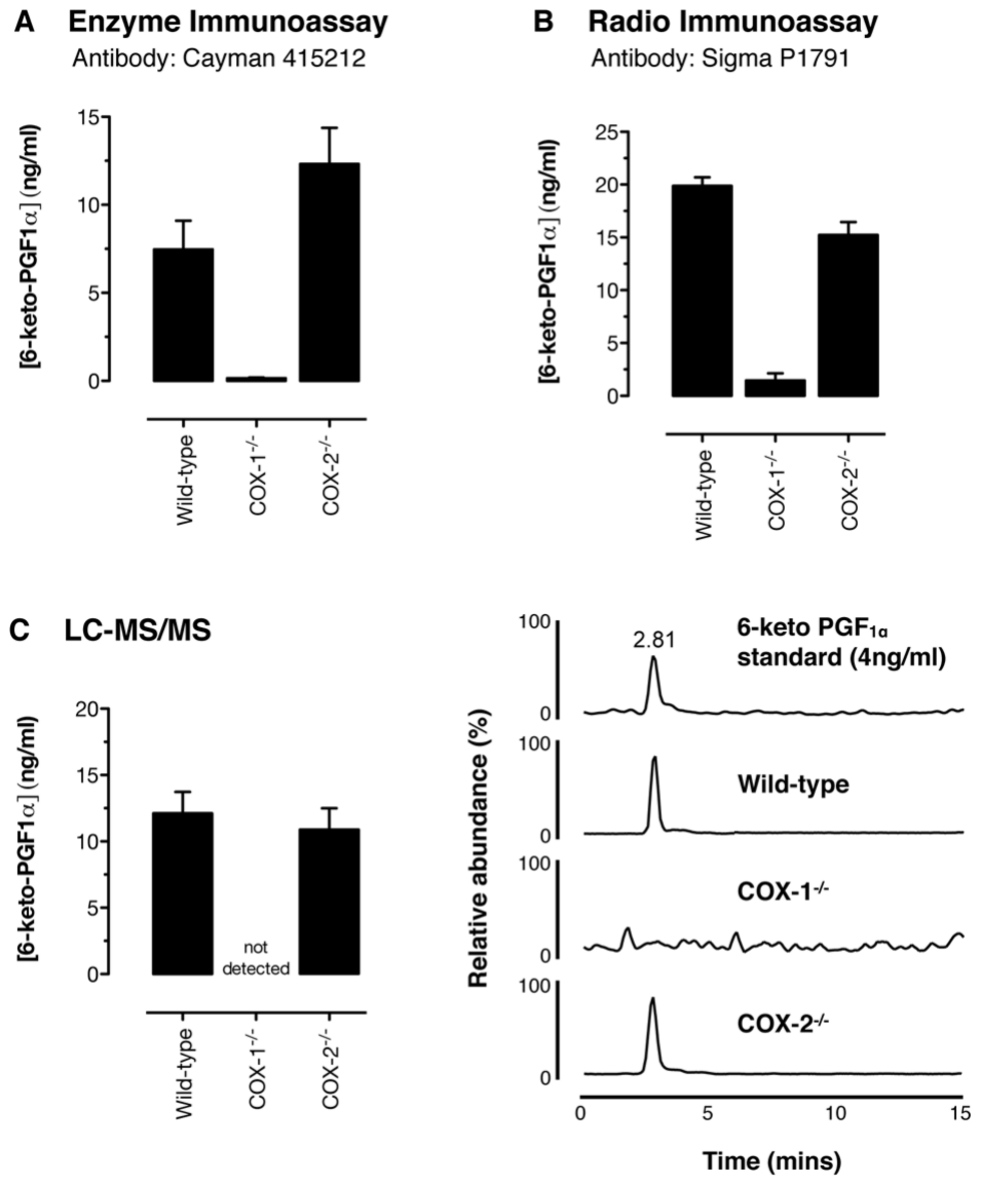
6-keto-PGF_1α_ production in isolated mouse aorta; measurement by enzyme immunoassay, radio immunoassay, and liquid chromatography tandem mass spectrometry (LC-MS/MS). Prostacyclin release by isolated rings of mouse aorta stimulated with Ca^2+^ ionophore A23187 (50µM), measured as the stable breakdown product 6-keto-PGF_1α_, was not altered by *Cox2* gene deletion, but was reduced >10-fold by *Cox1* gene deletion. The pattern and level of 6-keto-PGF_1α_ accumulation was similar whether measured by (a) enzyme immunoassay, (b) radio immunoassay or (c) LC-MS/MS. Representative LC-MS/MS chromatograms show the presence or absence of 6-keto PGF_1α_ in all sample types (retention time 2.81 min; transition ion *m*/*z* 369>163). n=4-7. *, p<0.05 by 1-way ANOVA with Bonferonni’s post-test.

Prostacyclin was discovered in the 1970s as a profoundly active hormone released by the blood vessel wall and readily detectable in experiments in which isolated blood vessels were activated and then mixed with bioassay systems such as platelets. After the structure of prostacyclin was elucidated, its stable breakdown products, including 6-keto-PGF_1α_, were identified. Antibodies to 6-keto-PGF_1α_ were subsequently raised, and immunoassays were developed [30]. 6-keto-PGF_1α_ immunoassays have since been widely used and have been instrumental in developing and expanding the field of prostacyclin biology. However, because they rely on antibody-antigen reactions, results with immunoassays can be confounded with artifacts, e.g., cross reactivity with related antigens and matrix interactions [16]. Here we measure vascular 6-keto-PGF_1α_ production using two different immunoassays, and validate these measurements with mass spectrometry (Figure 1). In each case, 6-keto-PGF_1α_ release by isolated aorta was readily detectable in tissue from wild-type and COX-2-deficient mice, but was undetectable (<0.2 ng/ml by our LC-MS/MS assay) in tissue from COX-1-deficient mice (Figure 1). Similarly, release of PGE_2_, 13,14-dihydro-15-keto-PGE_2_, PGD_2_, TXB_2_ and PGF_2α_ by aortic rings, measured by LC-MS/MS was in each case driven by COX-1 (Table 1).

**Table 1:** Prostanoid release by isolated aortic rings, measured by LC-MS/MS.

**Mediator**	**Wild-type**	***Cox1*^*-/-*^**	***Cox2*^*-/-*^**
	pg/ml	pg/ml	pg/ml
6-keto-PGF_1α_	12110 ± 1623	not detectable	10870 ± 1614
PGE_2_	1217 ± 168	not detectable	1011 ± 200
13,14-dihydro-15-keto-PGE_2_	40 ± 14	not detectable	39 ± 14
PGD_2_	385 ± 52	not detectable	316 ± 65
TXB_2_	443 ± 111	not detectable	406 ± 61
PGF_2α_	395 ± 62	not detectable	397 ± 63

Prostanoid release from Ca^2+^ ionophore A23187 (50µM)-stimulated aortic rings, measured by liquid chromatography tandem mass spectrometry, was almost abolished by *Cox1* gene deletion, but not substantially altered by *Cox2* gene deletion. Both the pattern and numerical values of 6-keto-PGF_1α_ levels measured by this method correlate closely with our previous data obtained using enzyme immunoassay. n=4

Using enzyme immunoassay, we performed additional experiments to confirm that 6-keto-PGF_1α_ production and release was partially dependent upon an intact endothelium and that the requirement of COX-1 for prostacyclin release was consistent when vessels were stimulated with a range of biological and experimental endothelium activators (Table S1). These data are entirely consistent with what we [5] and others [21] have recently published.

Although some recent studies have suggested a role for COX-2 in prostacyclin production by vascular cells [14] or vessels [15] in culture, it is important to point out that COX-2 activity is induced quite rapidly when these biological samples are placed in culture [5]. For this reason it is essential that experiments are carried out on fresh vessels and that observations showing COX-2 expression and activity, after even brief culture periods, be interpreted with caution.

### Role of COX isoforms in eicosanoid generation in vivo; analysis of circulating 6-keto-PGF_1α_ levels and additional prostanoids by LC-MS/MS

**Figure 2:**
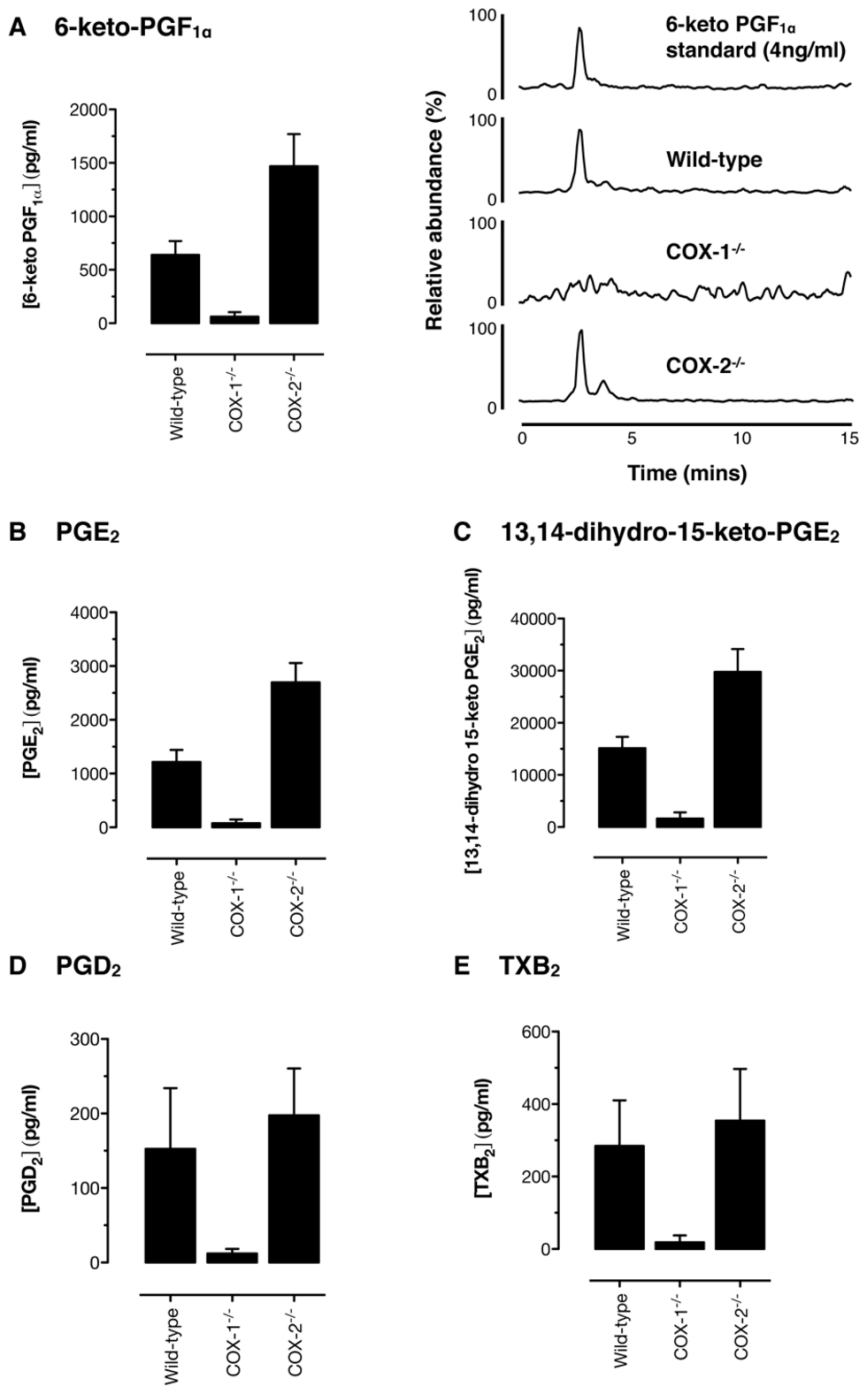
Bradykinin-stimulated prostanoid accumulation in the circulation *in* *vivo* in wild-type, *Cox1* ^*-/-*^, and *Cox2* ^*-/-*^ mice. Accumulation of the stable prostacyclin breakdown product, 6-keto-PGF_1α_ in plasma after bradykinin administration (100nmol/kg i.v.) is dependent on COX-1 but not COX-2 when measured by LC-MS/MS (a). Representative LC-MS/MS chromatograms show the presence or absence of 6-keto PGF_1α_ in all sample types (retention time 2.81 min; transition ion *m*/*z* 369>163). Similar data were obtained for plasma levels of PGE_2_ (b), 13,14-dihydro-15-keto-PGE_2_ (c), PGD_2_ (d), TXB_2_ (e) and (f) PGF_2α_. Plasma 6-keto-PGF_1α_ levels in all genotypes compare well with those previously published using enzyme immunoassay measurements. n=6. *, p<0.05 by 1-way ANOVA with Bonferonni’s post-hoc test.

Measuring markers of prostacyclin release from aortic vessels *in vitro* cannot tell us definitively what happens in the circulation *in vivo*. We previously demonstrated, using enzyme immunoassay, that both basal and bradykinin stimulated 6-keto-PGF_1α_ plasma levels were unaffected by *Cox2* gene deletion, but were greatly reduced by *Cox1* gene deletion [5]. Since experimental conditions could selectively influence the formation of 6-keto-PGF_1α_ [31], we have performed similar experiments and measured a panel of prostanoids in plasma by LC-MS/MS. Plasma levels of the prostanoids measured displayed the following rank order: 13,14-dihydro-15-keto-PGE_2_ >> PGE_2_^≈^ 6-keto-PGF_1α_ > TXB_2_^≈^ PGD_2_ in bradykinin-treated mice (Figure 2). In each case, plasma prostanoid levels were strongly reduced in Cox1^-/-^ mice, but not altered in Cox2^-/-^ mice. Plasma 6-keto-PGF_1α_ levels measured here by LC-MS/MS (Figure 2) closely matched those we previously reported using enzyme immunoassay measurement, a correlation recently suggested as necessary [16] to provide critical validation for our recent work [5].

### Tissue mapping both of expression from the Cox2 gene and of COX-2 bioactivity

Data in this paper, as well as previous work from our own [5] and other laboratories [21] firmly establishes COX-1 as the major COX isoform that drives vascular prostacyclin release in a healthy cardiovascular system. Importantly, this is also true in atherosclerosis; recent data from our group shows that COX-1 drives prostacyclin production even in segments of vessels heavily burdened with atherosclerosis [32]. Nevertheless, whilst COX-2 does not drive prostacyclin production, it clearly does impact on cardiovascular homeostasis; either inhibition of COX-2 activity or global *Cox2* gene deletion exacerbate atherosclerosis [13,32,33] and thrombosis [12,15] in mice, and the risk of atherothrombotic events is increased in patients taking drugs that inhibit COX-2 [34]. This conclusion leads to two important questions; (1) ‘if not in the arterial endothelium, where is COX-2 constitutively expressed?’ and (2) ‘how does COX-2 at sites remote from the vascular wall protect the cardiovascular system?’ Looking for COX-2 levels in organs and tissues using traditional immunohistochemical approaches relies on specificity and sensitivity of antibodies, with all the caveats and objections raised previously [16]. Comparison of COX-2 mRNA levels suffers from complications due to variability in extraction procedures, differences in mRNA stability in extracts, and clearly documented differences in transcription/translation coupling across cell types that results in COX-2 mRNA levels that do not reflect COX-2 enzyme activity. Here we have used a luciferase COX-2 reporter mouse (*Cox2* ^*fLuc/+*^) in which the firefly luciferase coding sequence is knocked into the *Cox2* gene at the start of site of translation of the endogenous COX-2 protein. Measuring luciferase expression allows rapid and reproducible visualization and quantitation of expression from the *Cox2* gene *in vivo* and *ex vivo* [27,35,36].

**Figure 3:**
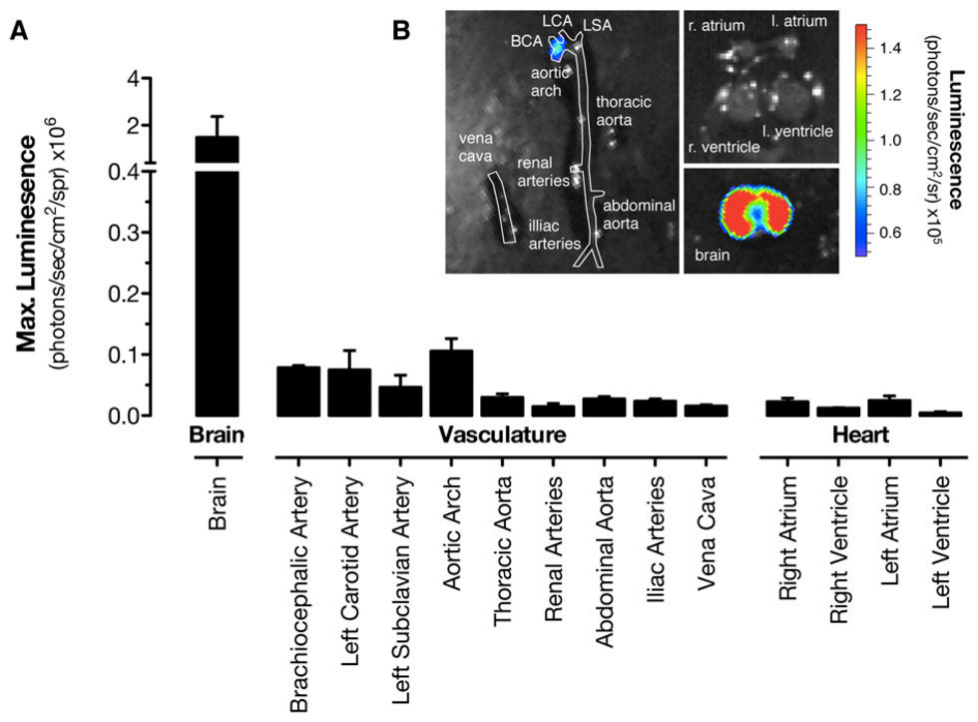
Distribution of luciferin-dependent bioluminescence in cardiovascular tissue from *Cox2* ^*fLuc/+*^ mice. (a) Quantification of basal expression from the aortic tree, vena cava, chambers of the heart and, for comparison, brain from *Cox2* ^*fLuc/+*^ mice and (b) and representative images of bioluminescence. Arteries, veins and chambers of the heart were essentially devoid of expression from the *Cox2* gene, in comparison with the brain as a reference tissue. The only exception to this was weak, but detectable, expression in the region of the aortic arch. n=3.

Using bioluminescent imaging of tissue dissected from *Cox2* ^*fLuc/+*^ mice, we first performed a systematic analysis of expression in tissues of the cardiovascular system. We imaged arterial expression in the entire aortic tree, as well as venous expression in the vena cava (Figure 3). As expected from our previous experiments, where COX-2 protein was measured using traditional antibody approaches [5], we found that the aorta was essentially devoid of *Cox2* gene expression (Figure 3) when compared to brain as a reference tissue [27]. The exception to this conclusion was the aortic arch and its branches, where low but detectable *Cox2* gene expression was found. Whilst this is consistent with the ‘priming’ of NFκB activity [37] and associated genes in this region of the aorta [38], it is important to put into context what this amount of *Cox2* gene expression actually means in terms of prostacyclin generation. Work from our previous study showed that prostacyclin release by mouse aortic arch was driven by COX-1, since activity was lost in arch tissue from Cox1^-/-^ mice but was unaffected in arch tissue from Cox2^-/-^ mice [5].

In addition to our observations on arterial and venous luciferase expression in *Cox2* ^*fLuc/+*^ mice, we performed specific sub-structural analysis of *Cox2* gene driven luciferase expression for each chamber of the heart (Figure 3), since conflicting results have been reported for the requirement for cardiomyocyte COX-2 expression in cardiac function [39,40]. *Cox2* gene expression was also essentially absent in each of the four chambers of the heart (Figure 3). These data on *Cox2* gene driven luciferase expression in the vasculature and heart confirm the sparse expression of the *Cox2* gene in the major structures of the cardiovascular system and fit precisely with immunohistochemistry and COX activity data both in this study and published recently by our group [5].

**Figure 4:**
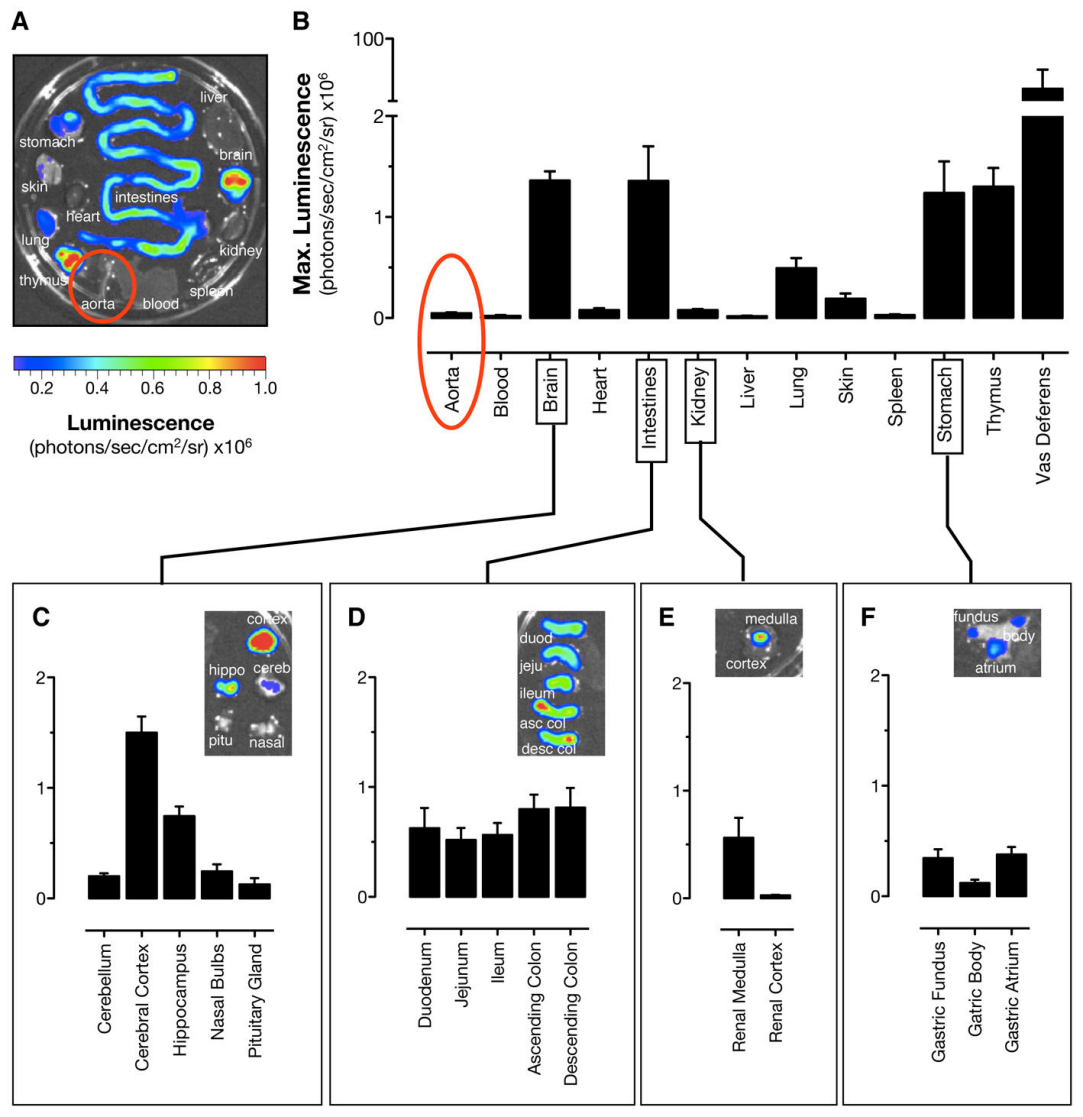
Distribution of luciferin-dependent bioluminescence in tissues from *Cox2* ^*fLuc/+*^ mice. (a) Basal expression from organs of the *Cox2* ^*fLuc/+*^ mice was visualized by bioluminescent imaging of tissues dissected from *Cox2* ^*fLuc/+*^ reporter mice after injection of D-luciferin in vivo (125mg/kg i.p.). (b) Imaging data are expressed as maximum luminescent emission from each tissue. Basal *Cox2* gene driven luciferase expression was present in many tissues including the vas deferens, brain, intestine, and thymus but was notably low to absent in the aorta (highlighted with red circles). Sub-division of the (c) brain, (d) intestine, (e) kidney and (f) stomach revealed regional expression patterns within each tissue. n=5.

**Figure 5:**
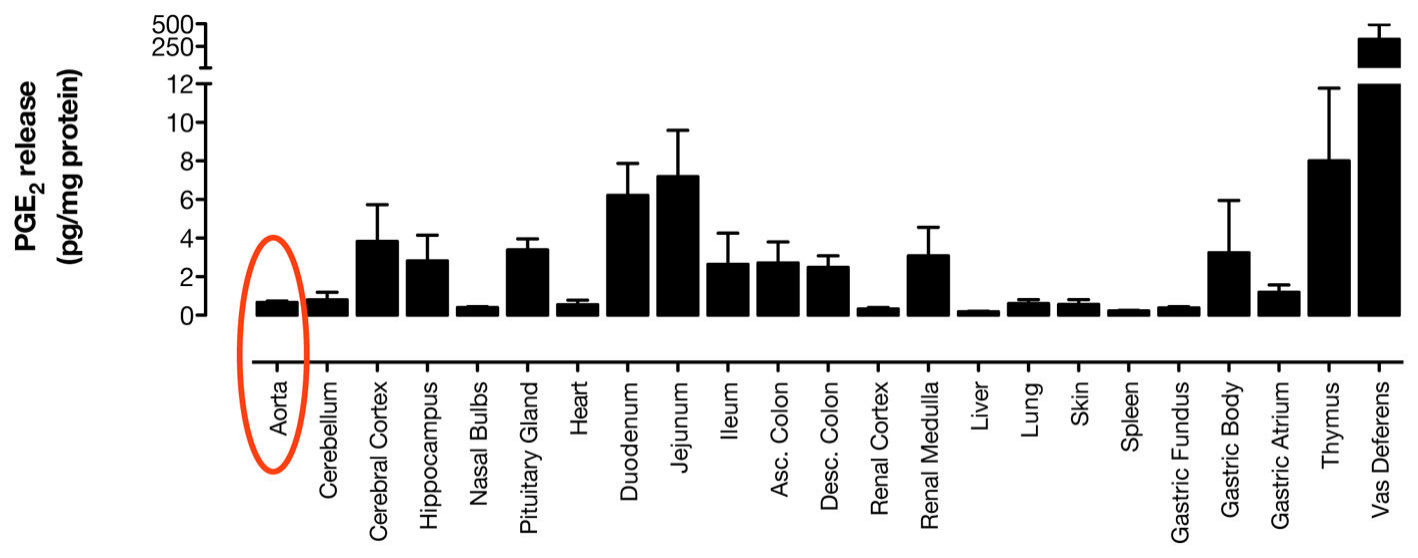
COX-2-dependent prostanoid production by aorta versus other mouse tissues in *Cox1* ^*-/-*^ mice. (a) PGE_2_ formation, normalized to tissue mass, was measured by immunoassay in supernatants of Ca^2+^ ionophore A23187 (50µM)-stimulated tissue segments from *Cox1* ^*-/-*^ mice. Cox1^-/-^ tissues released a variable amount of PGE_2_ with low levels in the aorta (highlighted in red), and substantially higher levels in the thymus, intestines, renal medulla, brain and vas deferens. This distribution correlates well with luciferase expression in organs of the *Cox2* ^*fLuc/+*^ mouse, as described in Figures 3 and 4. n=6.

With the very low to undetectable levels of *Cox2* gene expression in the heart and large vasculature confirmed, we next determined which organs *do* demonstrate substantial *Cox2* gene expression under normal homeostatic conditions. To do this we examined luciferase expression across a bank of organs from *Cox2* ^*fLuc/+*^ mice (Figures 4 and 5) and compared results with those from the aorta. The highest expression level from the *Cox2* gene occurred in the vas deferens (Figure 4), a result consistent with observations made by antibody-based methods for COX-2 protein quantification [27]. Substantial luciferase expression from the *Cox2* gene was also observed in the cerebral cortex, throughout the gastrointestinal tract, the thymus, and the renal medulla (Figures 4 and 5). Note that expression in each of these tissues was at least 10-fold greater than that in the aorta.

“Quantification” of luciferase activity by optical imaging of excised organs is only semiquantitative, because of light absorption by tissue, light scatter, and variability of luciferin substrate availability *in vivo*. However, precise quantification can be obtained by preparing tissue extracts and measuring light emission in saturating amounts of luciferin substrate. Quantification of luciferase activity in tissue homogenates from the *Cox2* ^*fLuc/+*^ mouse evaluated in Figure 4 reflected the same general pattern and activity of luciferase expression observed by optical imaging (Figure S1). Once again, in the context of this report, luciferase expression from the *Cox2* gene was essentially undetectable in the aorta.

In separate experiments we compared levels of Ca^2+^ ionophore-stimulated PGE_2_ release from tissue segments of Cox1^-/-^ mice, using PGE_2_ release as a first approximation of the relative COX-2 enzymatic activities in these tissues (Figure 5). COX-2-dependent PGE_2_ formation closely correlated with the pattern of luciferase expression in tissues from *Cox2* ^*fLuc/+*^ mice. COX-2 activity was highest in the thymus, gut, brain and vas deferens. However, consistent with the data in Figure 1, PGE_2_ was almost completely absent in the aorta. Comparison data for PGE_2_ production in tissues from wild-type and *Cox2* ^*-/-*^ mice are shown in Figure S2. Readers should note the difference in the scales for PGE_2_ values in Figure 5 versus Figure S2.

### Summary and Conclusions

Circulating prostacyclin (6-keto-PGF_1α_) and other prostanoids can be detected in mouse plasma using LC-MS/MS after bradykinin activation of the endothelium. The production of prostanoids found in the systemic circulation is driven overwhelmingly by COX-1 and not COX-2. The levels of 6-keto-PGF_1α_ measured by LC-MS/MS directly correlate with those we have previously observed by immunoassay, validating our previous observations and providing additional evidence for the absence of extensive COX-2-dependent prostacyclin formation in the circulation *in vivo*. In agreement, studies using *Cox2* ^*fLuc/+*^ reporter mice clearly demonstrate the absence of *Cox2* gene expression in blood vessels, but provide evidence for relatively high levels of constitutive COX-2 expression elsewhere, such as the thymus, brain, kidney and gastrointestinal tract. Taken together, these data not only provide additional confirmation for the absence of COX-2 expression and activity in the vasculature, but provide a systematic analysis of the distribution of *Cox2* gene expression throughout the body. We should now look more closely into the role of COX-2 expressed outside major blood vessels in explaining the adverse cardiovascular effects of COX-2 inhibition. This will allow us to move forward the development of novel prostaglandin-targeted therapies both for existing indications such as treatment of arthritis in patients with gastrointestinal compromise, as well as for emerging indications including cancer chemoprevention.

## Supporting Information

Figure S1 Distribution of luciferase activity in tissue homogenates from *Cox2^fLuc/+^* mice. Luciferase activity was determined quantitatively in homogenates of organs from *Cox2^fLuc/+^* reporter mice. As with bioluminescent imaging data, luciferase assays of homogenates in the presence of excess luciferin substrate confirmed the aorta (highlighted in red) to be essentially devoid of *Cox2* gene driven expression, whereas relatively high expression levels were present in brain, intestine and thymus. n=5. Luciferase activity was not determined (nd) in blood or vas deferens.
(PDF)

Figure S2 Total and COX-1-dependent prostanoid production by aorta versus other mouse tissues in wild type and *Cox2^-/-^* mice. PGE_2_ formation, normalized to tissue mass, was measured by immunoassay in supernatants of Ca^2+^ ionophore A23187 (50µM)-stimulated tissue segments from wild-type (a) and *Cox2^-/-^* mice (b). Prostanoid production patterns in each genotype illustrate that although tissues possess a variable amount of COX-2 activity, with the exception of the vas deferens, COX-1 is the dominant activity present. n=6.
(PDF)

Table S1 COX-1 and COX-2-dependent prostacyclin release both by endothelium-intact aorta and by endothelium-denuded aorta stimulated with a range of activators. Prostacyclin release, measured by enzyme immunoassay as 6-keto-PGF_1α_, was nearly abolished by *Cox1* gene deletion, but not by *Cox2* gene deletion, both in (a) endothelium-intact and (b) endothelium-denuded aortic rings. Reduction in 6-keto-PGF_1α_ production occurs both for basal release and for release stimulated by a range of endothelial activators. Prostacyclin release was attenuated by mechanical removal of the endothelium. n=6.
(DOCX)
