# Supplementary figures and images for: LC-MS/MS Confirms That COX-1 Drives Vascular Prostacyclin Whilst Gene Expression Pattern Reveals Non-Vascular Sites of COX-2 Expression
Source: PLoS One. 2013 Jul 9;8(7):e69524. doi: 10.1371/journal.pone.0069524 (PMC3711559; doi:10.1371/journal.pone.0069524)

# Luciferase Activity

(RLU/mg protein)  $\times 10^4$

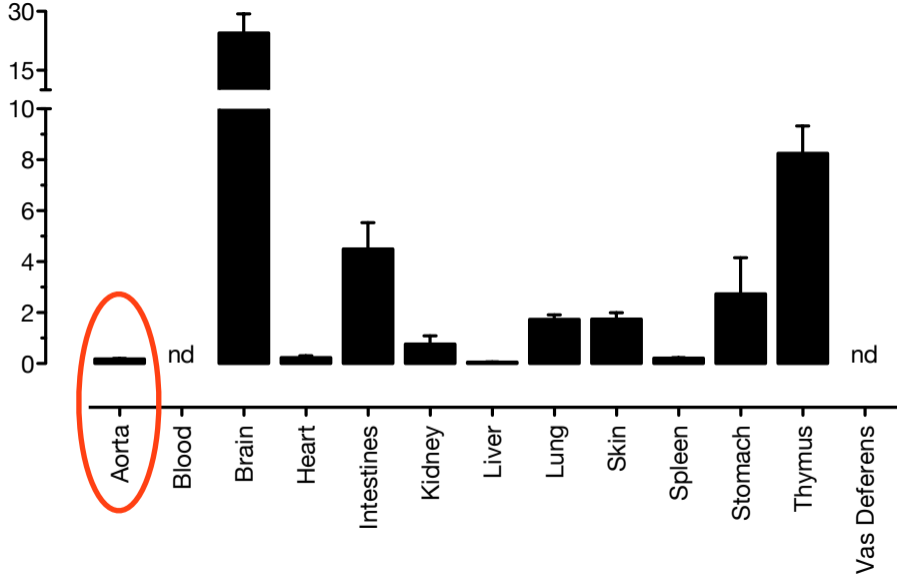

Supplement: Figure S1 — Luciferase activity was determined quantitatively in homogenates of organs from Cox2fLuc/+ reporter mice. As with bioluminescent imaging data, luciferase assays of homogenates in the presence of excess luciferin substrate confirmed the aorta (highlighted in red) to be essentially devoid of Cox2 gene driven expression, whereas relatively high expression levels were present in brain, intestine and thymus. n=5. Luciferase activity was not determined (nd) in blood or vas deferens. (PDF) [file pone.0069524.s001.pdf]

## A Wild-type

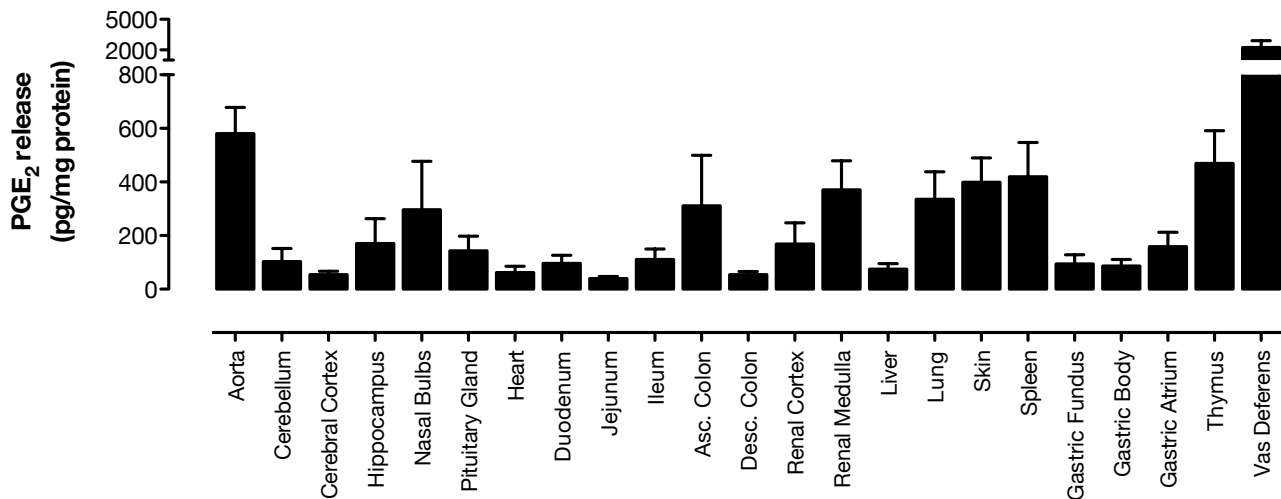

## B COX-2<sup>-/-</sup>

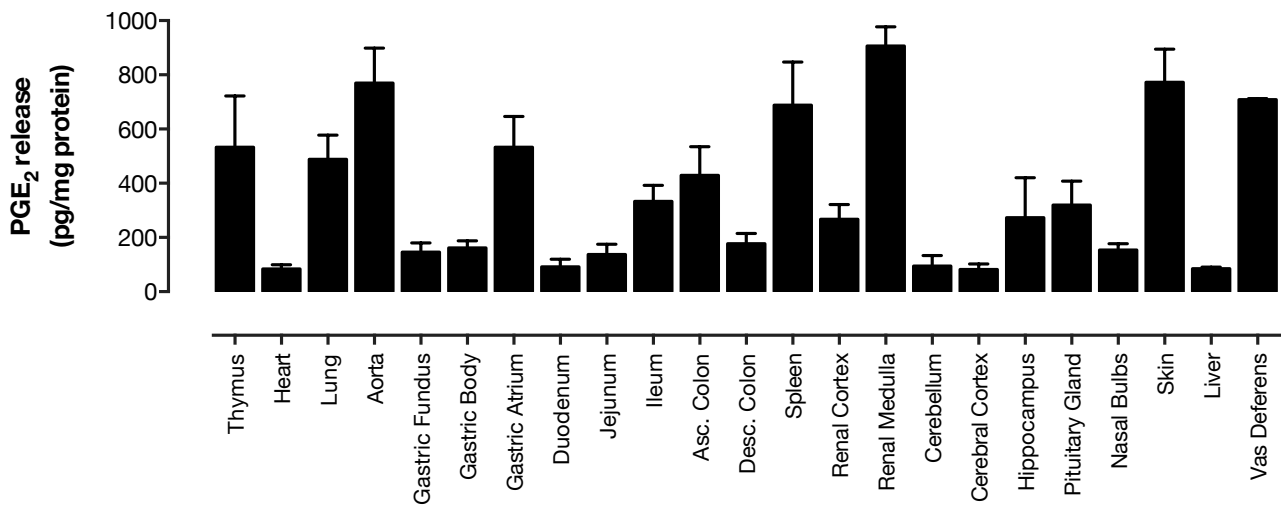

Supplement: Figure S2 — PGE2 formation, normalized to tissue mass, was measured by immunoassay in supernatants of Ca2+ ionophore A23187 (50µM)-stimulated tissue segments from wild-type (a) and Cox2-/- mice (b). Prostanoid production patterns in each genotype illustrate that although tissues possess a variable amount of COX-2 activity, with the exception of the vas deferens, COX-1 is the dominant activity present. n=6. (PDF) [file pone.0069524.s002.pdf]
