# Supplementary material for: LC-MS/MS Confirms That COX-1 Drives Vascular Prostacyclin Whilst Gene Expression Pattern Reveals Non-Vascular Sites of COX-2 Expression
Source: PLoS One. 2013 Jul 9;8(7):e69524. doi: 10.1371/journal.pone.0069524 (PMC3711559; doi:10.1371/journal.pone.0069524)
Supplement: Table S1 — Prostacyclin release, measured by enzyme immunoassay as 6-keto-PGF1α, was nearly abolished by Cox1 gene deletion, but not by Cox2 gene deletion, both in (a) endothelium-intact and (b) endothelium-denuded aortic rings. Reduction in 6-keto-PGF1α production occurs both for basal release and for release stimulated by a range of endothelial activators. Prostacyclin release was attenuated by mechanical removal of the endothelium. n=6. (DOCX) [file pone.0069524.s003.docx]

**Table S1. COX-1 and COX-2-dependent prostacyclin release both by endothelium-intact aorta and by endothelium-denuded aorta stimulated with a range of activators.**

| **Agonist** | **Wild-type** | ***Cox1^-/-^*** | ***Cox2^-/-^*** |
| --- | --- | --- | --- |
|  | [6-keto-PGF_1α_] ng/ml | [6-keto-PGF_1α_] ng/ml | [6-keto-PGF_1α_] ng/ml |
| (a) Endothelium Intact |  |  |  |
| Vehicle (0.1% DMSO) | 0.52 ± 0.26 | 0.02 ± 0.01 | 0.65 ± 0.19 |
| A23187 (50μM) | 7.47 ±1.63 | 0.15 ± 0.04 | 9.30 ± 1.17 |
| Bradykinin (100nM) | 0.69 ± 0.32 | 0.01 ± 0.01 | 0.70 ± 0.11 |
| Thrombin (1U/ml) | 0.78 ± 0.29 | 0.01 ± 0.00 | 0.48 ± 0.09 |
| ADP (10μM) | 0.23 ± 0.04 | 0.01 ± 0.00 | 0.41 ± 0.09 |
| Acetylcholine (10μM) | 1.22 ± 0.47 | 0.02 ± 0.01 | 1.59 ± 0.39 |
|  |  |  |  |
| (b) Endothelium Denuded |  |  |  |
| Vehicle (0.1% DMSO) | 0.28 ± 0.04 | 0.01 ± 0.00 | 0.29 ± 0.04 |
| A23187 (50μM) | 1.07 ± 0.41 | 0.07 ± 0.02 | 1.67 ± 0.84 |
| Bradykinin (100nM) | 0.37 ± 0.09 | 0.01 ± 0.00 | 0.47 ± 0.14 |
| Thrombin (1U/ml) | 0.51 ± 0.24 | 0.01 ± 0.00 | 0.41 ± 0.10 |
| ADP (10μM) | 0.36 ± 0.14 | 0.01 ± 0.00 | 0.31 ± 0.06 |
| Acetylcholine (10μM) | 0.28 ± 0.06 | 0.01 ± 0.00 | 0.59 ± 0.13 |
